# Regulating Global Sumoylation by a MAP Kinase Hog1 and Its Potential Role in Osmo-Tolerance in Yeast

**DOI:** 10.1371/journal.pone.0087306

**Published:** 2014-02-03

**Authors:** Ameair Abu Irqeba, Yang Li, Mahmoud Panahi, Ming Zhu, Yuqi Wang

**Affiliations:** 1 Department of Biology, Saint Louis University, St. Louis, Missouri, United States of America; 2 School of Medicine, Yunnan University, Kunming, Yunnan, China; Florida State University, United States of America

## Abstract

Sumoylation, a post-translational protein modification by small ubiquitin-like modifier (SUMO), has been implicated in many stress responses. Here we analyzed the potential role of sumoylation in osmo-response in yeast. We find that osmotic stress induces rapid accumulation of sumoylated species in normal yeast cells. Interestingly, disruption of MAP kinase Hog1 leads to a much higher level of accumulation of sumoylated conjugates that are independent of new protein synthesis. We also find that the accumulation of sumoylated species is dependent on a SUMO ligase Siz1. Notably, overexpression of *SIZ1* in *HOG1*-disruption mutants (*hog1Δ*) but not in wild type cells leads to a markedly increased and prolonged accumulation of sumoylated species. Examination of osmo-tolerance of yeast mutants that display either an increase or a decrease in the global sumoylation level revealed an inverse relationship between accumulation of sumoylated conjugates and osmo-tolerance. Further investigation has shown that many of the sumoylated species induced by hyperosmotic stress are actually poly-sumoylated. Together, these findings indicate that abnormal accumulation of poly-sumoylated conjugates is harmful for osmo-tolerance in yeast, and suggest that Hog1 promotes adaptation to hyperosmotic stress partially via regulation of global sumoylation level.

## Introduction

Hog1 is a yeast mitogen-activated protein kinase (MAP kinase, or MAPK) that is required for cell survival in response to increased osmolarity in the environment [1], [2]. Yeast cells can sense osmolarity changes via one of two transmembrane proteins, Sho1 and Sln1, which subsequently triggers a cascade of enzymatic events leading to the activation of Hog1 [1], [3]. Hog1 appears to be necessary for changes elicited by high osmolarity stimulation that are required for cells to adapt and survive in such conditions, as evidenced by the inability of the *hog1Δ* cells to grow in the presence of high osmolarity [4]. The mechanisms by which Hog1 promotes osmo-tolerance are under intensive investigation. It was thought that Hog1 exerts its function mainly via phosphorylating nuclear transcriptional regulators such as Hot1 to modulate gene expression [5], [6]. Notably, an elegant subsequent work suggested that transcriptional regulation may not be the key mechanism by which Hog1 promotes osmo-tolerance, as preventing nuclear entrance of Hog1, a necessary step for Hog1 to modulate gene transcription, does not affect the ability of Hog1 to promote osmo-tolerance [7]. Thus, it appears that Hog1 may exert its essential role in osmo-adaptation via additional mechanisms, such as controlling the nuclear export of mRNA upon stress [8].

Post-translational modification of proteins by small ubiquitin-like modifier (SUMO) has emerged as a common mechanism for cell regulation [9]. Similar to the process of ubiquitination, SUMO is conjugated to a lysine residue on substrate via the action of E1 SUMO-activating enzyme, E2 SUMO-conjugating enzyme, and in certain cases E3 SUMO-ligating enzyme [9]. In yeast, Ubc9 is the only E2 enzyme for sumoylation, while Siz1, Siz2 and Mms21 are three known E3 ligases for sumoylation [10]–[12]. Just as ubiquitin can form poly-ubiquitin chains, SUMO can also be conjugated to other SUMO to form chains. Three lysine residues at the N-terminal of yeast SUMO (i.e., K11, K15, and K19) are the sites for the formation of poly-SUMO chain in yeast [13]. Sumoylation is also a reversible modification. In yeast, Ulp1 and Ulp2 are the two known de-sumoylating enzymes [14], [15]. It is believed that Ulp1 is responsible for de-conjugating solitary SUMO proteins while Ulp2 is responsible for disassembling poly-SUMO chains [13].

Sumoylation has been implicated in stress responses in a variety of systems including yeast, plant seedlings and mammalian cells [16]–[19]. Various stress conditions, such as oxidative stress, ethanol stress, osmotic stress as well as heat shock, can drastically increase the accumulation of sumoylated conjugates [16]–[18]. However, the underlying molecular mechanism and whether sumoylation is critical for stress adaptation are largely unclear. Work in plants demonstrated a clear correlation between accumulation of SUMO1/2-conjugated proteins and impairment in salt-tolerance [17]. This suggests that accumulation of sumoylated proteins to an abnormal level could be detrimental to stress adaptation. Therefore, mechanisms must exist to protect cells under osmotic stress by preventing abnormal accumulation of harmful SUMO-conjugates.

In this study, we examined the involvement of sumoylation in the yeast response to osmotic stress. We show that osmotic stress induces rapid accumulation of sumoylated conjugates but the level of accumulation is limited by a stress-activated MAP kinase Hog1. We find Siz1 but not Siz2 is mainly responsible for the accumulation of osmo-induced SUMO conjugates. Interestingly, overexpression of the *SIZ1*gene leads to a drastically increased and prolonged accumulation of osmo-induced sumoylated species in the *hog1Δ* mutant. Consistent with the notion that abnormal accumulation of sumoylated conjugates is harmful for stress tolerance, we find overexpression of *SIZ1* exacerbates the sensitivity of the *hog1Δ* cells to hyperosmotic stress. Thus, our analysis reveals a new mechanism by which Hog1 promotes adaptation to osmotic stress, namely, via limiting the accumulation of harmful sumoylated conjugates induced by such stress.

## Materials and Methods

### Strains and Plasmids

Standard methods for the growth, maintenance, and transformation of yeast and bacteria and for the manipulation of DNA were used throughout. The yeast *S. cerevisiae* strains used in this study are BY4741 (*MAT* **a** *his3Δ leu2Δmet15Δura3Δ*), BY4741-derived mutants lacking *HOG1*, *SIZ1*, and *SIZ2* (Research Genetics, Huntsville, AL), BY4741-derived mutant carrying a catalytic inactive allele of *HOG1* (*hog1^K52R^*) (from Stephen Parnell at University of Kansas), BY4741-derived mutant lacking both *HOG1* and *SIZ1* (BY4741 *hog1::LEU2 siz1::KanMX*, this work), W303 strain MHY500 (*MAT* **a** *his3-Δ200 leu2-3,112 ura3-52 lys2-801 trp1-1*),MHY500-derived mutants harboring temperature-sensitive allele of *ubc9-1* (*MAT* **a** *his3-Δ200 leu2-3,112::LEU2::ubc9-1 ura3-52 lys2-801 trp1-1 ubc9Δ::TRP1*) and *ulp1-333* (*MAT* **a** *his3-Δ200 leu2-3,112::LEU2::ulp1-333 ura3-52 lys2-801 trp1-1 ulp1Δ1::his3::URA3*), MHY500-derived mutants lacking *ULP2* (*ulp2-Δ1::HIS3*) from Mark Hochstrasser at Yale [15], JD-52 strain (*MAT* **a** *trp1-Δ1 ura3-52 his3-Δ200 leu2-3,112 lys2-801*) and JD-52-derived mutants IS30 (*ulp2Δ::URA3*), GBY1 (*MAT* **a** *smt3-R11,15,19*::*TRP1*), GBY7 (*MAT* **a** *smt3-R11,15,19*::*TRP1 ulp2Δ*::*URA3*) from Erica Johnson at Thomas Jefferson University [13], and IS30-derived mutants lacking both *ULP2* and *HOG1* (*ulp2Δ::URA3 hog1Δ::LEU2*, this work). Plasmid overexpressing *SIZ1*, i.e., BG1805-*GAL-SIZ1*-TAP was purchased from OpenBiosystems.

### Growth, Phosphorylation, and Sumoylation Bioassays

Growth assays were conducted as described previously [20]. Phosphorylation of Hog1 was monitored by immunoblotting of whole cell extracts, using antibodies that recognize dually phosphorylated p38 [21]. For all the immunoblotting analysis, mid-log cell cultures were grown on appropriate medium, treated or not treated with 1 M sorbitol for indicated length of time. Growth was stopped by the addition of 10 mM NaN_3_ and transfer to an ice bath. Cells were washed and proteins were extracted via trichloroacetic precipitation, following procedures described previously [22]. Whole cell extracts were resuspended in boiling SDS-PAGE sample buffer (62.5 mM Tris-HCl, pH 6.8, 10% glycerol, 2% SDS, 1% 2-mercaptoethanol, 0.0005% bromphenol blue) for 5 min. Following SDS-polyacrylamide gel electrophoresis and transfer to nitrocellulose, the membrane was probed with antibodies to phosphor-p38 at 1∶1,000 (from Cell Signaling), and SUMO at 1∶10,000 (from Stefan Jentsch, Max Planck Institute of Biochemistry, Germany). Immunoreactive species were visualized by enhanced chemiluminescence detection (Pierce) of horseradish peroxidase-conjugated anti-rabbit IgG (Bio-Rad) or anti-goat IgG (Santa Cruz Biotechnology). Specificity of detection was established using *hog1Δ*, and *ubc9-1* cell extracts as negative controls. Note that an efficient detection of very high molecular weight species (on the very top of the gel) accumulated in the *ulp2Δ* mutant requires anti-SUMO antibody to be very fresh. All experiments have been repeated two or three times.

## Results

Hog1 is a MAP kinase in yeast that is primarily responsible for eliciting responses that ensure proper adaptation and survival of cells to hyperosmotic stress [1]. We were interested in understanding mechanisms by which Hog1 promotes adaptation to high osmolarity. Our objective here is to investigate whether Hog1 is involved in the regulation of global sumoylation. Previously, it has been shown in plants that salt treatment leads to accumulation of sumoylated proteins [17], [19]. Intriguingly, the degree of accumulation of sumoylated proteins seems to *inversely* correlate with the capability of the plant seedlings to tolerate salt stress [17]. This suggests that abnormal accumulation of sumoylated proteins could be detrimental to cells and mechanisms must exist to limit the accumulation of sumoylated proteins.

**Figure 1:**
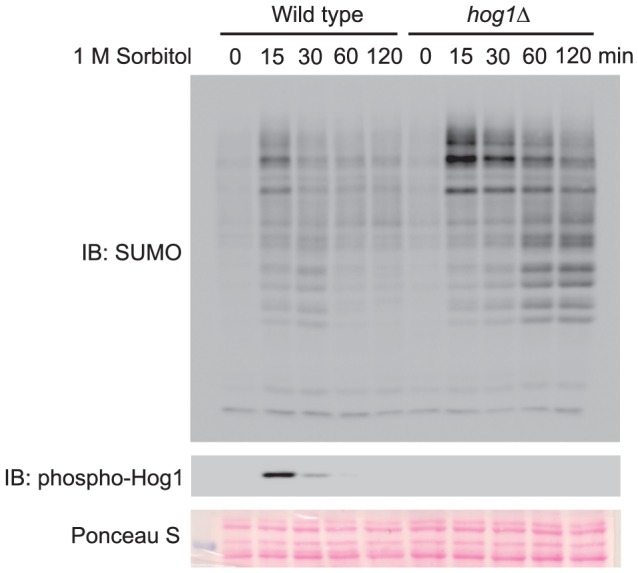
A MAP kinase Hog1 limits the accumulation of sumoylated conjugates induced by hyperosmotic stress. Whole cell extracts were prepared from wild type and *hog1Δ* mutants treated with 1 M sorbitol for the indicated time, resolved by 8% SDS-PAGE, and probed with anti-SUMO (*top panel*) or anti-phospho-Hog1 (*middle panel*) antibodies. The specificity of SUMO antibody was established by previous work and was further confirmed by cell extracts from SUMO E2-defective mutant cells (*data not shown*). The equal loading of each lane was confirmed by Ponceau S staining (*bottom panel*). IB, immunoblotting.

We hypothesize that signaling pathway evoked in response to hyperosmotic stress could be responsible for preventing accumulation of sumoylated proteins to a level that is detrimental to the cells. To test this hypothesis in yeast, we first determined whether hyperosmotic stress also induces accumulation of sumoylated conjugates in yeast. For this purpose, we examined the level of sumoylated conjugates in cells that were treated with 1 M sorbitol over time. Indeed, hyperosmotic stress induced a transient but substantial accumulation of sumoylated conjugates at the time point of 15 minutes, followed by a gradual decline of these conjugates (**Fig. 1**). Interestingly, the decline of sumoylated conjugates occurred right after the maximal activation of Hog1, a MAP kinase that is responsible for normal osmo-tolerance in yeast, as detected by anti-phospho-p38 that recognizes dually phosphorylated and thus activated Hog1 (**Fig. 1**, *lower panel*) [21], [23]. To determine whether Hog1 has any inhibitory role on the accumulation of sumoylated conjugates induced by hyperosmotic stress, we conducted similar experiment in a *hog1Δ* mutant. Remarkably, a much higher level of sumoylated conjugates is accumulated in the *hog1Δ* mutant, as compared to wild type cells **(** **Fig. 1** **)**. This suggests that Hog1 indeed has a role in limiting the accumulation of sumoylated conjugates triggered by hyperosmotic stress.

**Figure 2:**
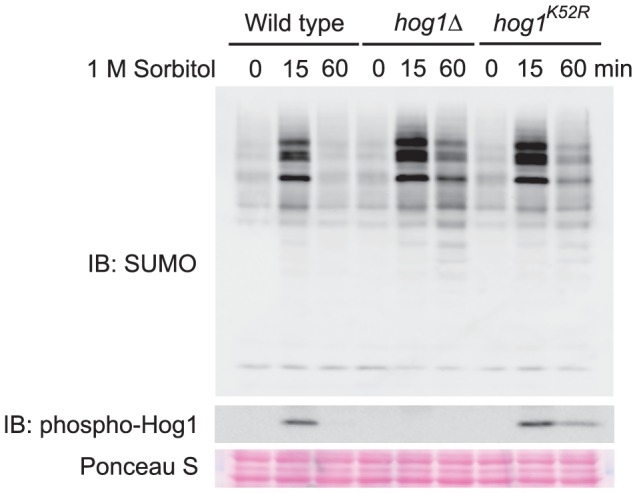
Catalytic activity of Hog1 is required for its inhibition of global sumoylation. Whole cell extracts were prepared from wild type, *hog1Δ*mutants, and *hog1^K52R^* mutants treated with 1 M sorbitol for the indicated time, resolved by 8% SDS-PAGE, and probed with anti-SUMO (*top panel*) or anti-phospho-Hog1 (*middle panel*) antibodies. The equal loading of each lane was confirmed by Ponceau S staining (*bottom panel*). IB, immunoblotting.

To determine whether the kinase activity of Hog1 is required in this process, we examined the behavior of *hog1^K52R^*, a catalytically inactive mutant of Hog1. In this mutant, a lysine residue of Hog1 that is responsible for binding ATP has been changed to an arginine. Consequently, this mutant can be phosphorylated by its upstream kinase Pbs2 but lacks the ability to phosphorylate other proteins. As reported previously, in response to sorbitol stimulation, the *hog1^K52R^* mutant displayed prolonged phosphorylation, compared to wild type, as detected by immunoblotting with anti-phospho-p38 antibody (**Fig. 2**, *middle panel*) [3], [23]. Similar to the *hog1Δ* mutants, a higher level of sumoylated conjugates is accumulated in the *hog1^K52R^* mutants, especially in the later time points (**Fig. 2**, *upper panel*). Thus, inhibiting the accumulation of osmo-triggered sumoylation requires the kinase activity of Hog1.

**Figure 3:**
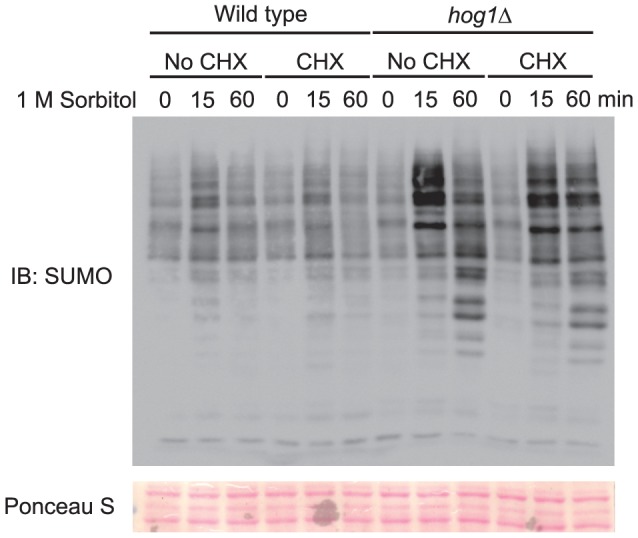
New protein synthesis is NOT required for Hog1 to prevent the accumulation of sumoylated conjugates induced by hyperosmotic stress. Whole cell extracts were prepared from wild type and *hog1Δ*mutants treated with protein synthesis inhibitor cycloheximide (CHX) to stop new protein synthesis, followed by 1 M sorbitol for the indicated time, resolved by 8% SDS-PAGE, and probed with anti-SUMO antibodies. The equal loading of each lane was confirmed by Ponceau S staining. IB, immunoblotting.

Many effects exerted by Hog1 are mediated via activation of its downstream transcription factors, which consequently leads to new gene transcription and new protein synthesis. It is possible that regulation of global sumoylation by Hog1 is mediated by its downstream transcription factors. In that case, we expect that new protein synthesis might be required for the inhibitory effect of Hog1. To test this, we repeated the experiments in wild type and the *hog1Δ* mutants, with or without the addition of protein synthesis inhibitor cycloheximide. As shown in **Fig. 3**, cycloheximide (CHX) treatment has nearly no effect on changes in the global sumoylation triggered by hyperosmotic stress, in either wild type or the *hog1Δ* mutants, especially in the later time points (i.e., 60 min). Thus, the effect of Hog1 on limiting the accumulation of osmo-induced sumoylation does not require new protein synthesis, therefore excluding the possibility that the effect is mediated via transcription factors controlled by Hog1.

**Figure 4:**
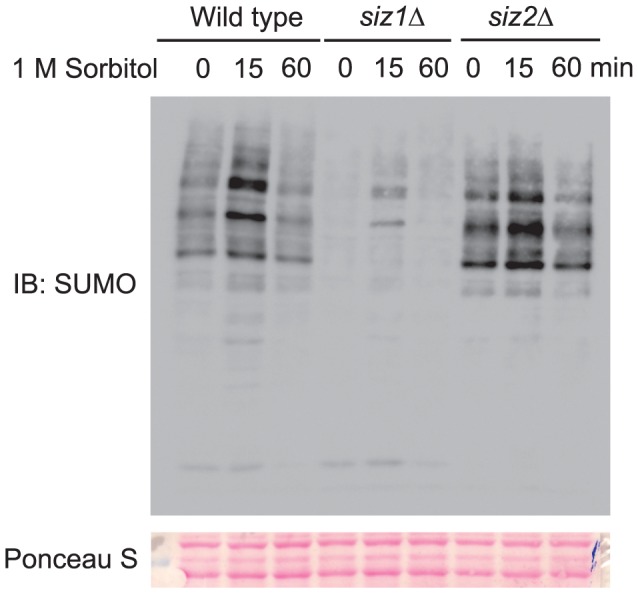
SUMO ligase Siz1 but not Siz2 is required for hyperosmotic stress-induced accumulation of sumoylated conjugates. Whole cell extracts were prepared from wild type, *siz1Δ*, and *siz2Δ* mutants treated with 1 M sorbitol for the indicated time, resolved by 8% SDS-PAGE, and probed with anti-SUMO antibodies. The equal loading of each lane was confirmed by Ponceau S staining. IB, immunoblotting.

Next, we consider the possibility that Hog1 regulates global sumoylation via modulating the activity of enzymes in the sumoylation pathway. To test this possibility, we sought to identify the ligase that is responsible for the observed accumulation of sumoylated conjugates. Siz1 and Siz2 are two well characterized E3 ligases that catalyze protein sumoylation in yeast [11]. To determine if they are responsible for hyperosmotic stress-induced sumoylation, we examined the sumoylation status of the *siz1Δ* and *siz2Δ* mutants, with or without hyperosmotic stress. As shown in **Fig. 4**, sumoylated species observed in wild type cells were largely absent in the *siz1Δ* but not the *siz2Δ* mutants. Thus Siz1 appears to be the main ligase that is responsible for the formation of sumoylation conjugates. The residual signals detected in the *siz1Δ* mutants indicate that additional ligase may also have a minor role in this process.

**Figure 5:**
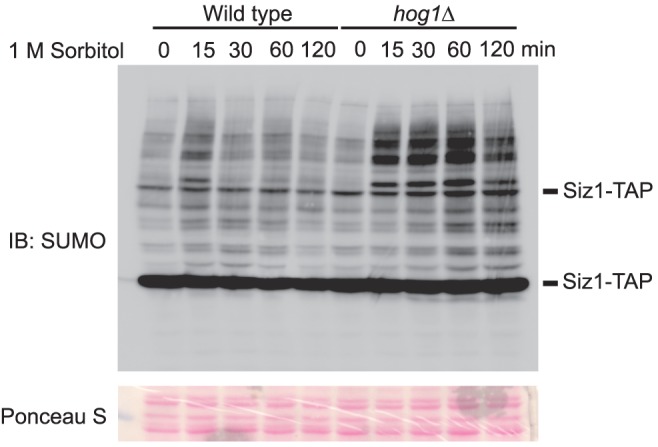
Overexpression of *SIZ1* in the *hog1Δ* mutants but not in the wild type increases hyperosmotic stress-induced sumoylation. Wild type or the *hog1Δ* cells were transformed with plasmids that overexpress TAP-tagged Siz1 (i.e., Siz1-TAP). Cells were grown to mid-log phase, treated with 1 M sorbitol for the indicated time, and harvested. Whole cell extracts were prepared and resolved by 8% SDS-PAGE, and probed with anti-SUMO antibodies. Note that TAP tag contains IgG binding motif, and thus Siz1-TAP was also recognized by SUMO antibody. For unknown reason, two bands with different sizes of Siz1-TAP were detected, and these two bands are absent in cells transformed with an empty vector.

Having identified Siz1 as the main ligase for the observed global sumoylation, we then proceeded to examine whether overexpression of *SIZ1* has any effect on the accumulation of sumoylated conjugates in hyperosmotic stressed cells. As shown in **Fig. 5**, overexpression of *SIZ1* in wild type cells has negligible effect on sumoylation (compare **Fig. 5** and **Fig. 1**). However, overexpression of *SIZ1* in the *hog1Δ* cells leads to a significantly enhanced and prolonged accumulation of SUMO-conjugates (**Fig. 5**). The drastically different outcomes of overexpressing *SIZ1* in wild type versus the *hog1Δ* mutants suggest that Hog1 has an inhibitory role on the activity of Siz1. As such, overexpressed Siz1 is rendered largely inactive in wild type cells. In contrast, the inhibitory effect of Hog1 on overexpressed Siz1 is relieved in the *hog1Δ* mutants, leading to an increased accumulation of SUMO-conjugates in osmotically stressed cells.

**Figure 6:**
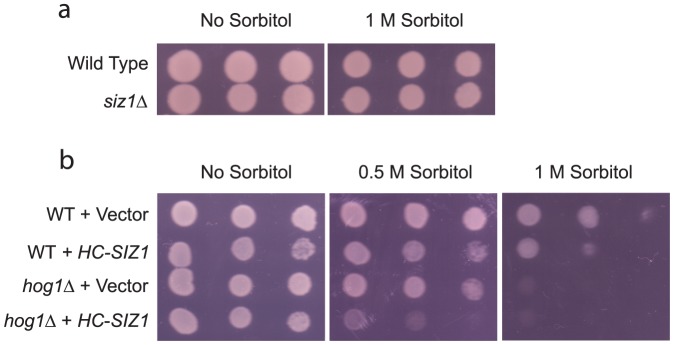
Overexpression of *SIZ1* enhances osmo-sensitivity of *hog1Δ* mutants. *a*, Osmo-sensitivity of wild type and *siz1Δ* mutants was measured by comparing the growth of serial diluted exponentially growing yeast cells on YPD plates with or without the addition of 1 M sorbitol. ***b***, wild type (WT) and *hog1Δ* mutants were transformed with either *URA3*-marked plasmids that overexpress *SIZ1* (i.e., *HC-SIZ1*) or *URA3*-marked empty vector. The osmo-sensitivity of the cells was measured by comparing the growth of serial diluted exponential cells on SCG-URA plates with or without the addition of various concentrations of sorbitol (0.5 M and 1 M, respectively).

Having identified situations that either diminish (i.e., *siz1Δ* mutants) or enhance (i.e., *hog1Δ* mutants, and overexpressing Siz1 in the *hog1Δ* mutants) osmo-induced accumulation of SUMO-conjugates, we then examined whether there is any correlation between the level of SUMO-conjugates and the ability of the cells to adapt to hyperosmotic stress. For this purpose, we compared the growth of these different cells with or without the addition of 1 M sorbitol in the plates. As shown in **Fig. 6a**, both wild type and the *siz1Δ* mutants grew equally well with or without the addition of 1 M sorbitol, suggesting that accumulation of SUMO-conjugates triggered by hyperosmotic stress is not required for normal adaptation. As expected [2], disrupting Hog1 (i.e., *hog1Δ* mutants) renders the cells sensitive to hyperosmotic stress (**Fig. 6b**). Interestingly, the osmo-sensitivity of the *hog1Δ* mutants is significantly exacerbated by overexpressing the *SIZ1* gene. This synergistic effect is especially evident when a mild hyperosmotic stress (e.g., 0.5 M sorbitol) was applied (**Fig. 6b**). These data suggest an *inverse* relationship between the level of SUMO-conjugates and the tolerance of cells to hyperosmotic stress.

**Figure 7:**
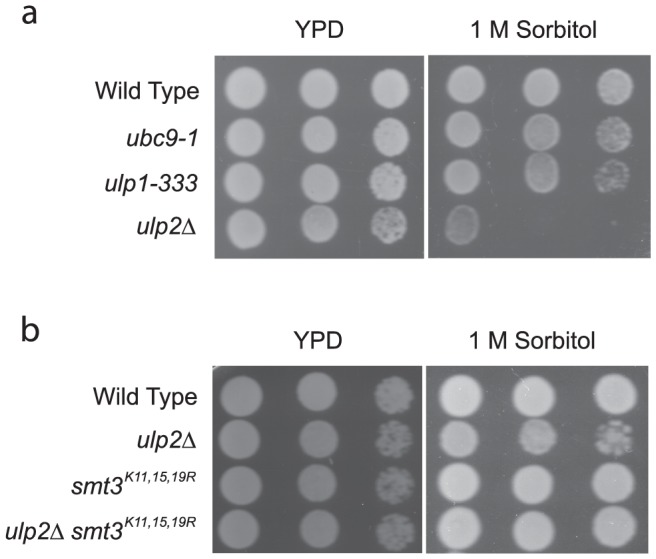
An accumulation of poly-sumoylated conjugates renders the *ulp2Δ* mutants more sensitive to hyperosmotic stress. *a*, osmo-sensitivity of wild type (MHY500) and MHY500-derived mutants *ubc9-1*, *ulp1-333* and *ulp2Δ*, was measured by comparing the growth of serial diluted exponentially growing yeast cells on YPD plates with or without the addition of 1 M sorbitol. ***b***, osmo-sensitivity of wild type (JD-52) and JD-52 derived mutants either accumulating poly-sumoylated species (*ulp2Δ*) or incapable of forming poly-sumoylated species (*smt3^K11,15,19R^* and *ulp2Δ*/*smt3^K11,15,19R^*) was similarly measured.

To further confirm a potential correlation between accumulation of SUMO-conjugates and osmo-intolerance, we also compared the growth of mutants lacking a functional Ubc9 (the sole E2 for sumoylation), Ulp1 (desumoylating enzyme), or Ulp2 (another desumoylating enzyme), with or without hyperosmotic stress. It has been shown previously that lacking a functional Ubc9 abolishes nearly all sumoylation events [10], while lacking a functional Ulp1 or Ulp2 leads to accumulation of SUMO-conjugates [14], [15]. As shown in **Fig. 7a**, similar to the *siz1Δ* mutants, lacking a functional Ubc9 has nearly no effect on the cell growth in the presence of 1 M sorbitol. Lacking a functional desumoylating enzyme Ulp1 has a modest effect on cell growth in the presence of 1 M sorbitol, while disrupting Ulp2 has a drastic effect. These results are consistent with our notion that sumoylation *per se* is not required for proper adaptation to hyperosmotic stress but abnormal accumulation of sumoylated species hinders the osmo-adaptation response.

It has been shown that Ulp1 and Ulp2 have distinct substrates. Specifically, disrupting Ulp1 leads to accumulation of mono-SUMO conjugated to substrates while disrupting Ulp2 leads to accumulation of poly-SUMO conjugates [13]. To test whether accumulation of poly-sumoylated species is responsible for the severe osmo-sensitivity of the *ulp2Δ* mutant, we examined whether blocking formation of poly-sumoylated species in the mutant can rescue its osmo-sensitivity. Poly-sumoylation can be prevented by mutating three lysine residues on SUMO that are necessary for chain formation (i.e., K11, K15, and K19) [24]. Accordingly, we compared the osmo-sensitivity of the *ulp2Δ* mutant and the *ulp2Δsmt3^K11, 15, 19R^* double mutant. As shown in **Fig. 7b**, the *ulp2Δsmt3^K11, 15, 19R^* double mutant grew much better than the *ulp2Δ* mutant in the presence of high osmolarity, suggesting that abnormal accumulation of poly-sumoylated species significantly contributes to the severe osmo-sensitivity of the *ulp2Δ* mutant.

**Figure 8:**
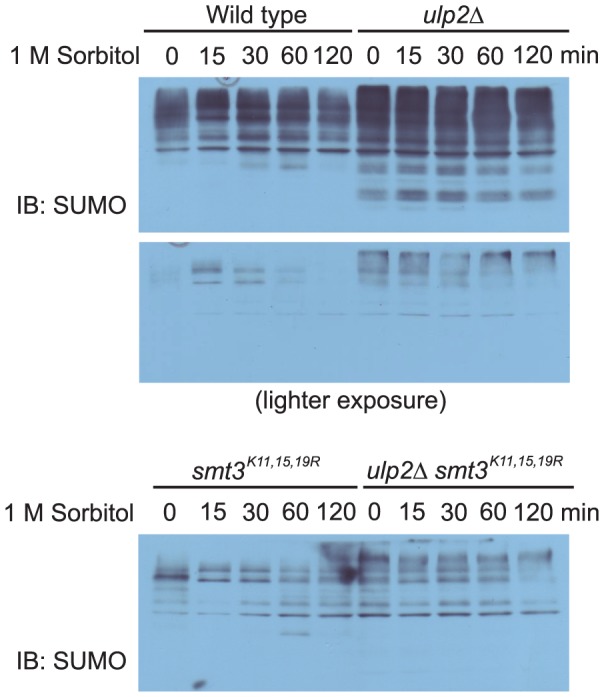
Hyperosmotic stress induces an accumulation of poly-sumoylated conjugates. Whole cell extracts were prepared from wild type (JD-52) and JD-52 derived mutants either accumulating poly-sumoylated species (*ulp2Δ*) or incapable of forming poly-sumoylated species (*smt3^K11,15,19R^* and *ulp2Δ*/*smt3^K11,15,19R^*) treated with 1 M sorbitol for the indicated time, resolved by 8% SDS-PAGE, and probed with anti-SUMO antibodies. IB, immunoblotting. Note that an efficient detection of the very high molecular weight species accumulated in the *ulp2Δ* mutants requires the antibody (anti-SUMO) to be very fresh.

We then examined whether hyperosmotic stress induces accumulation of poly-sumoylated species. For this purpose, we analyzed the global sumoylation level of wild type cells versus various mutants that either expresses mutant allele of SUMO or lacks *ULP2*, or both. As shown in **Fig. 8**, high molecular weight species were accumulated in the *ulp2Δ* mutants and mutating the three lysine residues to arginine as indicated drastically diminished the SUMO signal. Notably, hyperosmotic stress-induced change in global sumoylation is most prominent in wild type cells and such hyperosmotic stress-induced change is less apparent in the mutants (*ulp2Δ*, *smt3^K11, 15, 19R^* and *ulp2Δsmt3^K11, 15, 19R^*). These data indicate that sumoylated species induced by hyperosmotic stress are largely poly-sumoylated conjugates.

**Figure 9:**
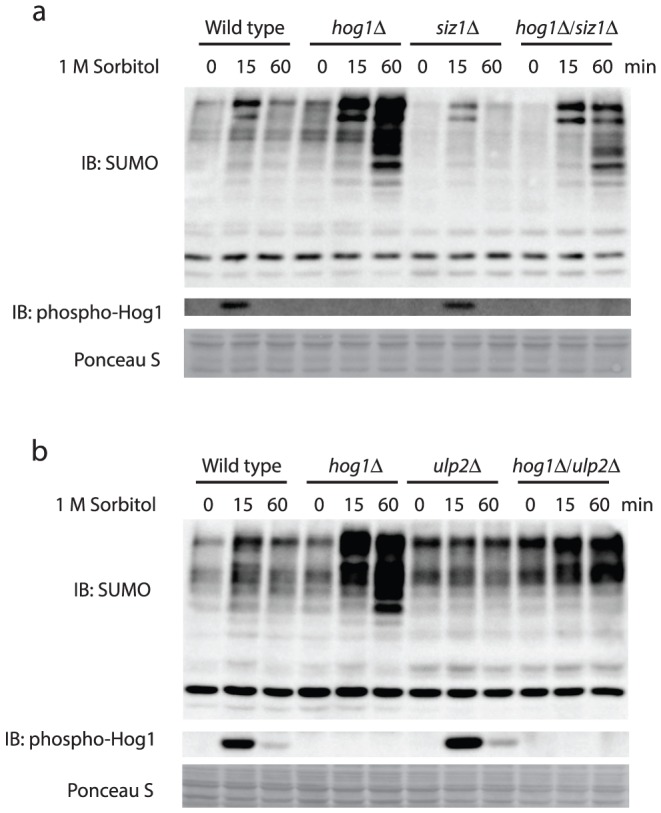
Both Siz1 and Ulp2 are required for Hog1 to limit accumulation of sumoylated conjugates induced by hyperosmotic stress. *a & b*, Whole cell extracts were prepared from wild type, *hog1Δ*, *siz1Δ*, *hog1Δ/siz1Δ*, *ulp2Δ*, and *hog1Δ*/*ulp2Δ* treated with 1 M sorbitol for the indicated time, resolved by 8% SDS-PAGE, and probed with anti-SUMO antibodies. IB, immunoblotting.

Finally, we examined whether Siz1 and/or Ulp2 are required for Hog1 to regulate hyperosmolarity-induced sumoylation. For this purpose, we constructed double mutants (*hog1Δsiz1Δ* and *hog1Δ ulp2Δ*) and examined whether deleting either *SIZ1* or *ULP2* abolishes the enhanced accumulation of sumoyalted species in the *hog1Δ* cells. As shown in **Fig. 9a**, the marked accumulation of poly-sumoylated species observed in the *hog1Δ*cells in response to hyperosmotic stress is substantially diminished in the *hog1Δsiz1Δ* double mutants. Thus, Siz1 is partially responsible for the further enhanced accumulation of poly-sumoylated species in the *hog1Δ*cells. Deleting *ULP2* also diminishes the level of accumulation of poly-sumoylated species in the *hog1Δ* cells (**Fig. 9b**). Together, these results indicate that both Siz1 and Ulp2 are required for Hog1 to regulate the optimal level of hyperosmolarity-induced poly-sumoylation.

## Discussion

Most cells have the capacity to cope with environmental stresses such as changes in osmolarity. In yeast, successful adaptation to hyperosmotic stress requires the action of Hog1, a stress-activated protein kinase. In this study, we provide evidence that Hog1 promotes adaptation to high osmolarity partially via preventing and/or removal of the harmful accumulation of sumoylated species. We find that hyperosmotic stress induces a transient increase in the level of global sumoylation, but blocking sumoylation itself has no or negligible effects on long term osmo-adaptation, indicating that sumoylation *per se* does not play any apparent positive roles in promoting adaptations to hyperosmotic stress. However, aberrant accumulation of such species renders cells incapable of coping with hyperosmotic stress, and Hog1 prevents this catastrophic event from happening by limiting the accumulation of such harmful sumoylated species. In addition, we find the sumoylated species induced by hyperosmotic stress are largely poly-sumoylated. Together, these findings suggest that abnormal accumulation of poly-sumoylated species is harmful for osmo-tolerance, and that Hog1 promotes adaptation to high osmolarity partially via regulating the level of global poly-sumoylation.

It is unclear why abnormal accumulation of poly-sumoylated species is harmful for osmo-tolerance. One well-established function of poly-sumoylation is serving as an alternative signal that targets substrate proteins for proteasomal degradation. In this SUMO-targeted protein degradation pathway, poly-sumoylated substrates are recognized by the E3 ubiquitin ligase Slx5/Slx8, which contains SUMO-interacting motif (SIM), and become ubiquitinated and subsequently degraded in the proteasome [25], [26]. In theory, it appears possible that abnormal accumulation of poly-sumoylated species could alter the half-lives of certain proteins that are crucial for appropriate osmo-adaptation. However, neither *slx5Δ*nor *slx8Δ*cells display any altered sensitivity to hyperosmotic stress (data not shown). In fact, a survey of a library of deletion mutants that lack each of the known or putative E3 ubiquitin ligases in yeast [27] did not reveal any that displays an altered sensitivity to hyperosmotic stress (data not shown). Therefore, poly-sumoylation likely affects osmo-adaptation independent of targeting proteins for degradation.

Another possibility is that abnormal accumulation of poly-sumoylated species may hinder appropriate gene expression induced by hyperosmotic stress that is required for adaptation. When challenged with hyperosmotic stress, yeast cells initially repress overall gene expression by altering chromatin structure and releasing most regulating proteins from chromatin [28]. This transient gene repression phase (lasting less than 5 minutes) is followed by the induction of hundreds of genes, many of which are under the control of Hog1-activated transcription factors such as Hot1 [29]–[31]. A recent study indicated that SUMO chains (possibly via poly-sumoylation of certain protein substrates) are important for the maintenance of chromatin structure and transcriptional repression of stress-induced genes [32]. It is conceivable that rapid up-regulation of poly-sumoylation could initially help pausing global gene expression as an immediate response to hyperosmotic stress; however, abnormal accumulation (as seen in the *hog1Δ* cells) could have a detrimental effect on the derepression of numerous stress responsive genes, thereby leading to a defect in long-term adaptation to hyperosmotic stress. The transient nature of the poly-sumoylation response points to an effort by the cell to limit extended global SUMO conjugation; furthermore, the carefully timed nature of this response also lends support to the fact that prolonged accumulation of poly-sumoylated species is detrimental to the cell's adaptation to osmotic-stress.

Our data clearly showed that the activity of Hog1 is required for the maintenance of normal poly-sumoylation level induced by hyperosmotic stress. What could be the molecular mechanism by which Hog1 regulates poly-sumoylation? Our findings that the Hog1 effect is independent of new protein synthesis suggest that the regulation is not mediated via activation of transcription factors such as Hot1 that are under the control of Hog1. One possibility is that Hog1 directly regulates the activity of enzymes in the sumoylation pathway such as Siz1 and Ulp2, given that both proteins are required for Hog1 to limit the level of poly-sumoylation. However, neither Siz1 nor Ulp2 contains a preferred Hog1 phosphorylation site (PXS/TP), and phos-tag analysis of these two proteins did not reveal any obvious Hog1-dependent mobility shift. Thus, it is unlikely that Hog1 regulates these two enzymes via direct phosphorylation. Our initial attempt to detect a potential interaction between Hog1 and these two enzymes (Siz1 and Ulp2) was also not successful, suggesting either the interaction is too weak or too transient for detection, or that Hog1 regulates the activity of those enzymes indirectly. Future studies will be directed to elucidate the molecular mechanisms by which Hog1 regulates sumoylation pathway.

Our genetic analysis indicates that deleting either *SIZ1* or *ULP2* can partially suppress the accumulation of sumoylated species in the *hog1Δ* mutants. It is easy to understand why deleting *SIZ1* can suppress the accumulation of sumoylated species in the *hog1Δ* mutants because *SIZ1* encodes a sumoylating enzyme that is responsible for hyperosmotic stress-triggered sumoylation. Why does deleting *ULP2* also suppress the accumulation of sumoylated species in the *hog1Δ* cells, given that *ULP2* encodes a desumoylating enzyme? One possibility is that Ulp2 may have a role in regulating the abundance of Siz1. A very recent report has shown that the abundance of Siz1 can be regulated by SUMO-targeted ubiquitination pathway [33]. In this pathway, poly-sumoylation of the substrate acts as a signal for its subsequent ubiquitination and degradation [26]. Given the role of Ulp2 in reversing poly-sumoylation, Ulp2 may desumoylate Siz1 and thereby rescue Siz1 from ubiquitination and degradation by the SUMO-targeted ubiquitination pathway. Consequently, deleting *ULP2* may speed up the degradation of Siz1, which could result in suppressing the accumulation of sumoylated proteins in the *hog1Δ* mutants. This and other possibilities will be investigated in the future.
